# The MET Receptor Tyrosine Kinase Confers Repair of Murine Pancreatic Acinar Cells following Acute and Chronic Injury

**DOI:** 10.1371/journal.pone.0165485

**Published:** 2016-10-31

**Authors:** Ivana Gaziova, Daniel Jackson, Paul J. Boor, Dwayne Carter, Zobeida Cruz-Monserrate, Cornelis J. Elferink, Aditya D. Joshi, Bhupendra Kaphalia, Craig D. Logsdon, Karen Pereira de Castro, Lynn Soong, Xinrong Tao, Suimin Qiu, Lisa A. Elferink

**Affiliations:** 1 Department of Neuroscience and Cell Biology, University of Texas Medical Branch, Galveston, TX, United States of America; 2 Department of Pathology, University of Texas Medical Branch, Galveston, TX, United States of America; 3 Department of Pharmacology and Toxicology, University of Texas Medical Branch, Galveston, TX, United States of America; 4 Department of Microbiology and Immunology, University of Texas Medical Branch, Galveston, TX, United States of America; 5 Division of Gastroenterology, Hepatology and Nutrition, The Ohio State University Wexner Medical Center, Columbus, OH, United States of America; 6 Department of Cancer Biology, University of Texas MD Anderson Cancer Center, Houston, TX, United States of America; University of Szeged, HUNGARY

## Abstract

Acinar cells represent the primary target in necroinflammatory diseases of the pancreas, including pancreatitis. The signaling pathways guiding acinar cell repair and regeneration following injury remain poorly understood. The purpose of this study was to determine the importance of Hepatocyte Growth Factor Receptor/MET signaling as an intrinsic repair mechanism for acinar cells following acute damage and chronic alcohol-associated injury. Here, we generated mice with targeted deletion of MET in adult acinar cells (MET^-/-^). Acute and repetitive pancreatic injury was induced in MET^-/-^ and control mice with cerulein, and chronic injury by feeding mice Lieber-DeCarli diets containing alcohol with or without enhancement of repetitive pancreatic injury. We examined the exocrine pancreas of these mice histologically for acinar death, edema, inflammation and collagen deposition and changes in the transcriptional program. We show that MET expression is relatively low in normal adult pancreas. However, MET levels were elevated in ductal and acinar cells in human pancreatitis specimens, consistent with a role for MET in an adaptive repair mechanism. We report that genetic deletion of MET in adult murine acinar cells was linked to increased acinar cell death, chronic inflammation and delayed recovery (regeneration) of pancreatic exocrine tissue. Notably, increased pancreatic collagen deposition was detected in MET knockout mice following repetitive injury as well alcohol-associated injury. Finally, we identified specific alterations of the pancreatic transcriptome associated with MET signaling during injury, involved in tissue repair, inflammation and endoplasmic reticulum stress. Together, these data demonstrate the importance of MET signaling for acinar repair and regeneration, a novel finding that could attenuate the symptomology of pancreatic injury.

## Introduction

Pancreatitis is an excruciating and debilitating disease with no available treatments. The primary cause of pancreatitis involves acinar cell endoplasmic reticulum stress and/or the premature activation of pancreatic digestive enzymes in acinar cells, causing the loss of pancreatic acinar cells. Recent findings, however, challenge the latter paradigm [1]. The disease initially presents as an acute attack characterized by abdominal pain, nausea and/or vomiting. Repeated bouts of acute pancreatitis (AP) produce a persistent inflammatory response that can rapidly progress to chronic disease characterized by fibrosis, with long-term consequences including increased risk of diabetes or pancreatic cancer [2].

Tissue injury and inflammation are critical processes for tissue remodeling. However, failure to resolve these responses can lead to the destructive complications of chronic inflammation. Mouse models of pancreatic injury revealed the remarkable capacity of the exocrine pancreas for regeneration [3,4]. Acute pancreatic injury induced by the cholecystokinin analogue cerulein, causes increased acinar nuclear factor-ĸB (NF-ĸB) signaling with subsequent leukocyte recruitment [5]. Increased intrapancreatic inflammation amplifies the severity of injury, resulting in acinar cell death with induction of a regenerative response [6,7]. However, a detailed understanding of the upstream receptor signaling pathways guiding injury-associated acinar repair is far from complete.

The Hepatocyte Growth Factor Receptor (MET) is a tyrosine kinase that is known to participate in inflammatory responses and is critical for the self-renewing capability of stem cells in several cancers [8,9]. MET is typically expressed by epithelial cells and activated in a paracrine manner by binding Hepatocyte Growth Factor (HGF) [10]. The protective effects of MET signaling in monocyte-macrophage activation [11], B cell homing to the lymphoid microenvironment [12] and modulation of dendritic cell functions including migration, deactivation and immunoregulation [13] have been noted in various animal models of inflammatory disease, including arthritis, autoimmune inflammation and colitis [8]. However, the intrinsic ability of MET signaling to promote tissue repair and/or regeneration differs significantly between cell types and by the form of injury. For example, mice lacking MET in hepatocytes were hypersensitive to Fas-mediated injury consistent with an anti-apoptotic role [14]. Conversely, MET knockout in β islet cells caused reduced plasma insulin levels that were not associated with changes in islet mass, proliferation or morphology [15]. Keratinocytes lacking MET could not re-epithelialize skin wounds suggesting a cell migration defect [16]. Serum HGF levels are elevated in patients with AP, with significantly higher levels detected in severe cases with organ dysfunction [17]. The increase in serum HGF levels in patients with AP may reflect a self-defense mechanism important for tissue repair [18]. A specific role for MET signaling for acinar cell survival and/or regeneration in damaged pancreatic tissue remains unexplored.

In the present study, we investigated the role of MET for regeneration of the exocrine pancreas following cerulein-induced acute and recurrent (repetitive) acute injury, alcohol-associated chronic injury and alcohol-associated chronic injury in combination with repeated episodes of acute injury. In the normal adult pancreas, MET expression is low. We show elevated MET levels in ductal and acinar cells in human chronic pancreatitis (CP) specimens as well as transient upregulation of MET in the exocrine pancreas of cerulein-treated mice, consistent with a role for MET as an adaptive repair mechanism. We generated a novel, conditional MET knockout model in which MET is specifically deleted in mature acinar cells. Using this model we show increased acinar cell death, inflammatory infiltrates with edema in the pancreata of MET^-/-^ mice following two models of cerulein-induced injury–acute and repetitive acute injury. Furthermore, several inflammatory, macrophage and endoplasmic reticulum stress markers were markedly upregulated in MET^-/-^ pancreas under these conditions. The requirement for MET was not exclusive to acinar regeneration following cerulein-induced injury, as we found increased acinar cell death, chronic inflammation with collagen deposition in MET^-/-^ mice following chronic alcohol intake. Finally, we identified several novel targets downstream of acinar MET important for endoplasmic reticulum stress response, tissue inflammation and collagen deposition whose expression is altered in response to recurrent injury and chronic alcohol-associated injury, particularly in a mouse model of alcohol dependent pancreatic fibrosis. Our data support an important role for MET signaling for acinar regeneration and repair following acute and chronic injury.

## Materials and Methods

### Genetically engineered tissue-specific knockout mice

All animals were cared for in accordance with the Office for Protection from Research Risks (OPRR) and Animal Welfare Act guidelines under an animal protocol approved by the University of Texas Medical Branch Animal Care and Use Committee (IACUC). This study was approved by the University of Texas Medical Branch IACUC. Experimental MET^-/-^ animals were generated by crossing MET^fl/fl^ mice [19] with Bac-Ela-CreErT mice [20]. MET^fl/fl^, Bac-Ela-CreErT and MET^-/-^ strains were maintained on the C57BL/6J background. Littermate MET^fl/fl^ or wild-type C57BL/6J mice were used as controls. Acinar-specific knockout of MET was induced by tamoxifen treatment of 5–9 week old male mice (75 mg/kg) by oral gavage for three consecutive days and used one week following tamoxifen treatment. Cre-mediated MET knockout was confirmed by PCR using the following forward and reverse primers sets for MET (^5’^AGCTGGTCCAAGCAGTTCAG^3’^, ^5’^CAGGACCACCAGAGGAGAC^3’^) and CRE recombinase (^5’^CGGTCTGGCAGTAAAAACTAT^3’^, ^5’^CAGGGTGTTATAAGCAATCCC^3’^).

### Animal treatments

For mild acute cerulein-induced damage, age-matched male mice (N = 5/group) received seven hourly spaced intraperitoneal injections of cerulein (Bachem) at (50 μg/kg) dissolved in Dulbecco's Phosphate-Buffered Saline (DPBS) with 0.1% BSA or saline (control). Mice were euthanized at the indicated times following the last cerulein injection. For recurrent cerulein-induced damage age-matched male mice (N = 5/group) received six hourly spaced intraperitoneal injections of cerulein or saline, on alternate days three times. Mice were euthanized 72 h following the last cerulein injection. For alcohol-feeding studies, male mice (N = 3-4/group) were housed individually and sensitized to a Liber-DeCarli ethanol diet (LDC-E: diet #L10016, Research Diets, New Brunswick) ad libitum for two weeks, and sustained on a diet containing 3.5% ethanol for up an additional 14 weeks. During weeks 7, 9 and 11, two cohorts of mice were injected with 7 hourly spaced cerulein injections (50 μg/kg), three times per week. Individual animal weights and food intake were recorded daily. Animals were intraperitoneally injected with 100 μg/kg BrdU (Sigma) in phosphate buffered saline (PBS) 24 h prior to the euthanasia.

### Blood and tissue collection

Mice were sacrificed by cardiac puncture with cervical dislocation, blood was clotted for 1 h at room temperature and centrifuged (2,000xg/15 min/4°C) to obtain serum. Pancreas and other tissues were removed, dissected and portions stored or processed for RNA or protein analysis, and immunohistochemistry. For RNA analysis a ~2x2 mm^3^ piece of pancreas was immediately stored in RNAlater (Qiagen) solution at 4°C for RNA isolation. The remaining pancreatic tissues were halved and frozen in liquid nitrogen for protein analysis or fixed in 10% neutral buffered formalin for the histological analysis. Human pancreas samples were obtained from discarded pancreatic tissue following surgical resection, in accordance with the policies and practices of the Institutional Review Board of the University of Texas Medical Branch.

### RNA isolation and quantitative RT-PCR

Total pancreatic RNA was isolated using the guanidinium thiocyanate-cesium chloride [21]. Total RNA from other tissues was extracted using TRIzol (Life Technologies). RNA samples were treated with DNase I (Invitrogen) and used in qRT-PCR analyses with the following gene-specific forward and reverse primers: αSMA (NM_007392) ^5’^CCTCATGCCATCATGCGTC^3’^, ^5’^CAATCTCACGCTCGGCAGTA^3’^; COL1A1 (NM_007742) ^5’^CCTCCTGACGCATGGCC^3’^, ^5’^ATACAGATCAAGCATACCTCGGG^3’^; COL1A2 (NM_007743) ^5’^TCTGTCCTAGTCGATGGCTGC^3’^, ^5’^CAGAGGTGCAATGTCAAGGAAC^3’^; COL3A1 (NM_009930) ^5’^AAGGATGGAGAGTCAGGAAGACC^3’^, ^5’^CCATTGCGTCCATCAAAGC^3’^; EMR1 (NM_010130) ^5’^CCTCATTCACTGTCTGCTCAACC^3’^, ^5’^GAAGTCTGGGAATGGGAGCTAAG^3’^; HGF (NM_010427) ^5^TGATAAAGGAAAGTTGGGTTCTTACTG^3’^, ^5’^TCGCCTCTCTCATGAACATCG^3’^;IL1β (NM_008361) ^5’^TATGAGCTGAAAGCTCTCCACCTC^3’^, ^5’^GTCGTTGCTTGGTTCTCCTTG^3’^; RelA (NM_009045) ^5’^CTGTGCCTACCCGAAACTCAAC^3’^, ^5’^GGTTTGAGATCTGCCCTGATG^3’^; sXBP1 (NM_001271730 ^5’^GCTGAGTCCGCAGCAGG^3’^, ^5’^CAGAATGCCCAAAAGGATATCAG^3’^; TGFβ1 (NM_011577) ^5’^CCTTCCTGCTCCTCATGGC^3’^, ^5’^CGCACACAGCAGTTCTTCTCTG^3’^; TNFα (NM_013693) ^5’^CTCATCAGTTCTATGGCCCAGAC^3’^, ^5’^TCCTCCACTTGGTGGTTTGC^3’^. Primers for CHOP were designed according to Oslowski and Urano (2011) [22]. All qRT-PCR measurements were performed in triplicate and normalized to 18S values (^5’^ CGGCTACCACATCCAAGGAA^3’^, ^5’^GCTGGAATTACCGCGGT^3’^), and data are expressed as fold change relative to the controls.

### Antibodies

Antibodies against the following were used for IHC: BrdU (1:1000; abcam), insulin (1:1000; Cell Signaling), Ki67, a cellular marker for proliferation (1:3000; abcam), Ly6G, a marker for neutrophils (1:80; abcam), isolated macrophages of mouse origin (1:100, Santa Cruz, sc-101447), mouse MET (1.8 μg/ml; R&D), human MET (1 μg/ml; R&D). The following dilutions were used for western analysis: α-amylase (1:200000; Cell signaling), β-actin (1:10,000; Sigma) and mouse MET (0.2 μg/ml; R&D).

### Histology and Immunofluorescence

Paraffin embedded human pancreatic sections were deparaffinized, rehydrated and subjected to antigen retrieval in modified citrate buffer, pH 6.1 (DAKO) in a steamer for 20 min. Slides were treated with 3% H_2_O_2_ for 10 min to block endogenous peroxidase activity, washed with PBS and blocked in 10% horse serum for one hr. Samples from normal (N = 13) or CP (N = 16) patients were stained with human MET antibody (1 μg/ml; R&D) overnight and counterstained with Harris Hematoxylin (Statlab). Stained sections were graded blind by two individuals for MET staining intensity (MSI) in acinar and ductal cells as: 0 = baseline, 1 = low, 2 = medium or 3 = high.

Formalin-fixed mouse pancreatic sections were processed as described above and analyzed using MetaMorph software (Molecular Devices) [23] as follows with multiple microscopic fields (a minimum of 20). Edema was graded using a scale of 1 to 4, where 1 = 0–1%, 2 = 1–5%, 3 = 5–10%, 4 = 10%+ of pancreatic edema per high-power field (HPF) at 400x magnification. DNA fragmentation was assessed using terminal deoxynucleotidyl transferase-mediated deoxyuridine triphosphate nick end labeling (TUNEL) (In Situ Cell Death Detection Kit, TMR red, Roche). Cell nuclei were counterstained with DaPi to exclude false positive staining. Cell death was calculated as the number of TMR red and DaPi-positive cells per HPF at 100x magnification. Proliferation index was calculated as the number of BrdU or Ki67-positive cells per HPF at 200x magnification. Neutrophil infiltration was evaluated as the amount of Ly6G-positive cells per HPF at 200x magnification and scored as: 1 = no positive staining, 2 = 1–5 cells, 3 = 6–15 cells, and 4 = more than 16 cells. Macrophage infiltration was quantified as the percentage of Macrophage Marker-positive area per HPF at 100x magnification and scored as 1 = 0–0.5%, 2 = 0.5–3%, 3 = 3–6%, 4 = 6%+. Sections were graded blind by two individuals for MET staining intensity (MSI) as described above. Sirius Red staining was used to detect intrapancreatic collagen (NovaUltra Sirius Red Stain Kit; IHCWorld).

### Pancreatic protein extracts and western analysis

Pancreatic tissue samples were homogenized and analyzed by SDS-PAGE and Western blot analysis and the resulting digitized blots were quantified using MetaMorph software as previously described [23,24].

### Blood and serum analysis

Blood ethanol measurements were performed using 50 μl of whole heparinized blood in GC auto sampler vials as previously described [25]. A standard curve was generated from mixed known concentrations of ethanol. Serum Insulin was measured using Rat/Mouse Insulin Elisa kit (EMD Millipore). Active lipase in collected serum was measured using a Lipase Assay kit (IBL-America).

### Statistical analysis

Statistical significance was determined by Student’s t-test, 1-way ANOVA with a Dunnett’s post hoc analysis or a multiple t-test with Holm-Sidak correction for multiple analysis as indicated, using GraphPad Prism 6 (GraphPad Software). P<0.05 was considered statistically significant. Data are presented as a mean value ± SEM.

## Results

### MET is increased in human CP samples

**Fig 1:**
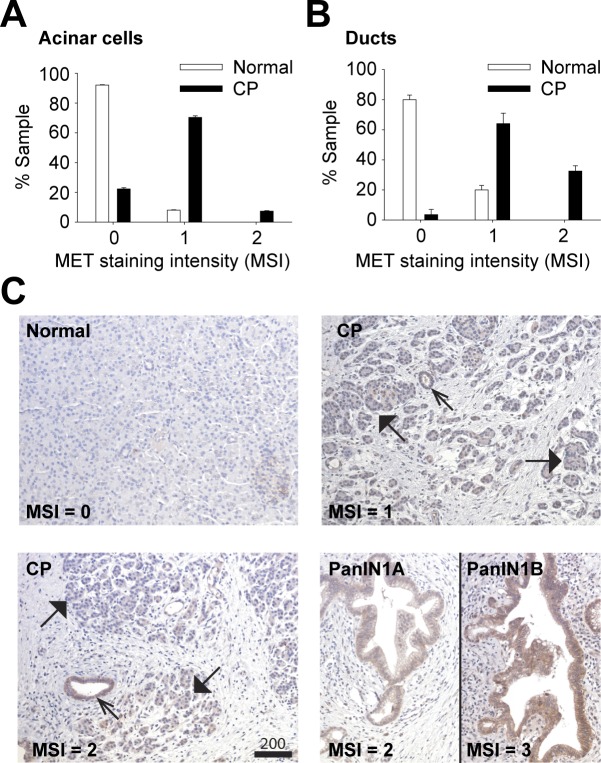
MET levels are increased in human CP. Pancreatic samples from normal (n = 13) or CP patients (n = 16) were H&E and MET stained. MET levels in acinar cells (A) and ducts (B) were scored as MET Staining Intensity (MSI) using a semi quantitative scale (0–3) where 0 = baseline, 1 = low, 2 = medium, 3 = high MET levels (Scale, microns). C, representative images are shown for normal, CP and PanINs. MET-positive ducts (open arrows) and acini (closed arrows) are indicated.

Serum HGF levels are known to be elevated in CP patients and are positively correlated with disease severity [18]. Accordingly, we anticipated increased MET levels in CP, consistent with a regenerative function for MET in the injured pancreas. CP patient samples were stained for MET expression and scored histologically in a blinded study. MET expression was significantly increased in the acinar (Fig 1A and 1C) and ductal cells (Fig 1B and 1C) of CP patients with expression higher in pancreatic intraepithelial neoplasia (PanIN) 1A and 1B lesions (Fig 1), established precursors to invasive pancreatic adenocarcinoma. MET staining was negligible in normal patient samples (Fig 1C).

### MET levels transiently increase in a mouse model of cerulein-induced acinar injury

**Fig 2:**
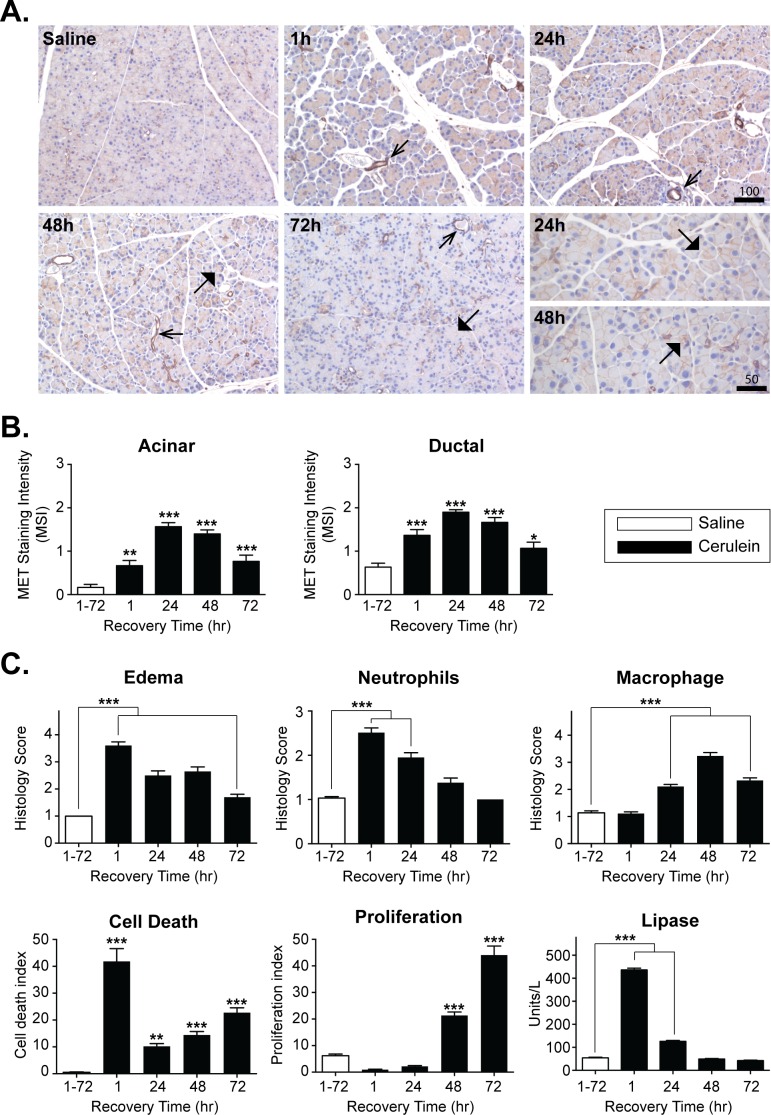
MET levels transiently increase in a mouse model of cerulein-induced AP. A, Mice were treated with cerulein or saline as a control, were sacrificed immediately 1 h after the last IP injection (1 h) or allowed to recover for 24–72 h following treatment. Pancreatic sections were processed for H&E and MET staining in ducts and ductal-like structures (open arrowheads) and acinar cells (closed arrowheads) (Scale, microns). Higher magnification showing increased MET staining in acinar membranes at 24 h and 48 h is shown. B, MET levels in acinar cells (Acinar) and ducts (Ductal) were scored as MET Staining Intensity (MSI) as described above. C, Morphometric analyses of edema, neutrophil and macrophage infiltration, acinar cell death and proliferation, and serum lipase levels are presented as mean ± SEM values from 4–5 mice per time point (one way ANOVA, *, p < 0.05; **, p < 0.01; ***, p < 0.001).

Acinar cells constitute the primary site of injury in CP [1,3,4]. The increased acinar MET expression associated with human CP may represent an intrinsic repair mechanism supporting the regeneration of acinar tissue. We detected a comparable change in MET expression using an established mouse model for acute acinar injury and regeneration (Fig 2). Repeated intraperitoneal (i.p.) administration of cerulein, a decapeptide analogue of the pancreatic secretagogue cholecystokinin, induces a mild reversible form of acinar cell injury in mice. A randomized cohort of C57BL/6J mice, were subjected to seven hourly i.p. injections of saline or cerulein (50 μg/kg/injection), and sacrificed either 1 h following the last injection or allowed to recover for up to 72 h. Pancreatic sections were analyzed for changes in MET expression, pancreatic edema, macrophage and neutrophil infiltration, acinar cell death and proliferation (Fig 2 and S1 Fig). In saline treated adult pancreas, MET levels were typically low in acinar cells. However, elevated acinar and ductal cell MET expression was evident 1 h following the last cerulein injection and peaked at 24 h before returning towards baseline by 72 h post treatment (Fig 2A and 2B). Coincident with the transient change in MET expression, prominent interstitial edema with neutrophil infiltration and high TUNEL staining was observed at 1 h following cerulein treatment (Fig 2C). The levels of edema and neutrophil infiltrates declined during the recovery period, whereas the cell death was biphasic, transiently peaking immediately after cerulein treatment before returning to near baseline by 24 h, followed by a secondary moderate increase over the ensuing 48 h. Increased macrophage infiltration and proliferation of surviving acinar cells peaked at 48 h and 72 h, respectively, in response to cerulein. Plasma lipase levels were increased 8–9 fold at 1 h following the final dose of cerulein, but returned to the baseline by 48 h (Fig 2C and S1 Fig**)**.

### Acinar-specific deletion of MET

**Fig 3:**
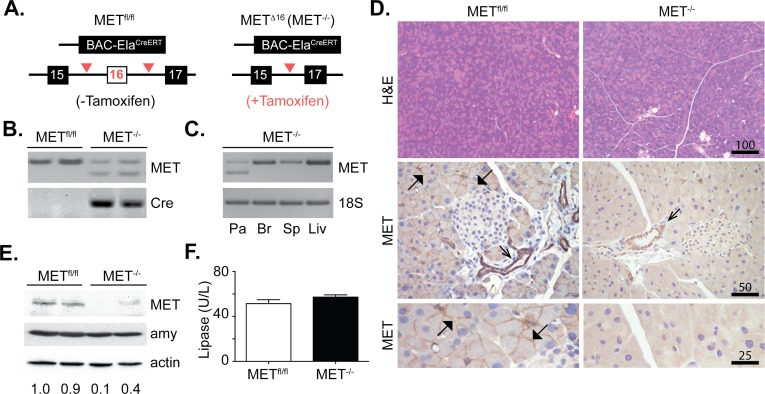
Conditional MET deletion in pancreatic acinar cells. A, Schematic analysis of the MET^fl/fl^ versus MET^-/-^ alleles. B, PCR analysis confirmed MET knockout, in Cre-expressing MET^-/-^ mice but not MET^fl/fl^ mice. C, PCR analysis confirmed pancreatic (Pa) MET knockout, but not in Brain (Br), Spleen (Sp) or Liver (Liv). 18S RNA served as a loading control. D, Representative H&E and MET staining in ducts and acinar membranes is shown (open and closed arrowheads respectively) (Scale, microns). E, Western analysis confirmed reduced MET protein expression in MET^-/-^ mice. Amylase (amy) and actin were unaffected. F, Serum lipase levels were unaffected in MET^fl/fl^ and MET^-/-^ mice. Data collected from 4–5 mice/ group.

MET is expressed by multiple cell types in the pancreas including acinar, islet, ductal and pancreatic progenitor cells, as well as leukocytes that rapidly infiltrate the pancreas in response to injury [7,26]. To evaluate the role for MET in acinar regeneration, it is necessary to disrupt MET activity during the regenerative response. MET inhibitors lack the fidelity to specifically attribute acinar MET signaling for acinar cell survival and regeneration following injury. Thus we genetically eliminated MET in adult acinar cells by crossing MET^fl/fl^ mice with a pancreatic acinar specific CreErT line (BAC-Ela-CreErT) under the control of the elastase I gene promoter. The Cre recombinase was activated in differentiated and adult acinar cells in response to tamoxifen (Fig 3A). Accordingly, we avoided anticipated complications associated with MET deletion during pancreatic development. The resulting acinar specific MET^-/-^ mice were viable and fecund, with no overt physical abnormalities. RT-PCR confirmed the expected 81-bp deletion in the MET transcript in the pancreas 7 days following tamoxifen (Fig 3B and 3C). H&E staining of pancreatic tissue from MET^fl/fl^ mice and MET^-/-^ mice showed well-organized pancreatic acini (Fig 3D) in both genotypes. Immunohistochemistry (IHC) detected MET expression in the ducts of MET^fl/fl^ and MET^-/-^ mice. MET staining was clearly evident in the acinar cells of MET^fl/fl^ mice but not in acinar cells of MET^-/-^ mice, consistent with an acinar cell-specific, Cre-mediated MET deletion (Fig 3D). Similarly, western analyses detected reduced MET protein in pancreatic protein extracts from MET^-/-^ mice (Fig 3E). We detected no difference in plasma lipase levels in the naïve pancreata of MET^fl/fl^ and MET^-/-^ mice (Fig 3F). The size and number of islets as well as serum insulin levels were directly comparable between MET^fl/fl^ and MET^-/-^ groups (S2 Fig).

### Loss of acinar MET expression increases the severity of cerulein-induced injury

**Fig 4:**
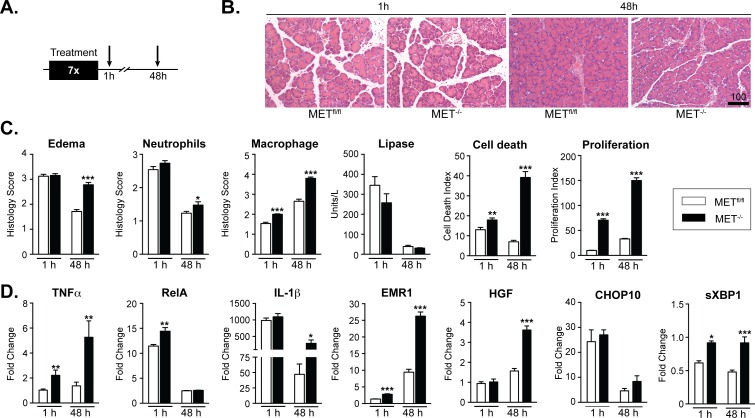
Genetic deletion of MET in acinar cells increased the severity of acute cerulein-induced injury. A, cerulein or saline treated MET^fl/fl^ and MET^-/-^ mice were sacrificed 1 h or 48 h following the last treatment. B, Representative H&E stained pancreatic sections, 1h and 48h following cerulein treatments are shown (Scale, microns). C, Morphometric analyses of edema, neutrophil and macrophage infiltration, acinar cell death and proliferation, and serum lipase levels are presented as mean ± SEM values from 4–5 mice per time point (multiple *t*-test,*, p < 0.05;**, p < 0.01; ***, p < 0.001). D, qRT-PCR confirmed relative changes in transcript levels in cerulein treated MET^fl/fl^ and MET^-/-^ mice for mediators of inflammation TNFα, RelA, Il-1β, macrophages (EMR1), HGF and ER stress markers (CHOP10 and sXBP1) (multiple t-test,*, p < 0.05;**, p < 0.01; ***, p < 0.001).

Hyperstimulation with cerulein, induces an acute and reversible form of pancreatitis in rodents [3,27]. Using the cerulein model for AP, we examined the importance of MET signaling in acinar injury and regeneration in MET^-/-^ versus MET^fl/fl^ mice. Mice were subjected to seven hourly i.p. injections of cerulein (50 μg/kg/injection) or saline and sacrificed either 1 h or 48 h following the last injection (Fig 4A). We selected time points at which MET levels are typically elevated and acinar tissue exhibits the features associated with acute injury (1 h) or early signs of recovery in wild-type mice (48 h) (See Fig 2). We reasoned that if MET signaling conferred immediate cytoprotection following acute injury, the acinar tissue in MET^-/-^ mice would display more severe damage 1 h following the last cerulein treatment, including increased edema and serum lipase, with enhanced cell death and granulocyte infiltration. In contrast, evidence of a role for MET activity in prolonged acinar cell survival and/or regeneration assumes that acinar cell death, inflammatory infiltrates and edema would persist in MET^-/-^ mice for 48 h. Pancreatic sections were analyzed for histological changes in tissue injury and repair. While the amount of edema was equivalent between the MET^-/-^ and MET^fl/fl^ mice immediately after the injury, edema was far more pronounced in the MET^-/-^ mice by 48 h (Fig 4B and 4C). The level of serum lipase and extent of neutrophil infiltration were equivalent between MET^-/-^ and MET^fl/fl^ mice immediately following cerulein injury suggesting that loss of MET does not enhance immediate tissue injury. Serum lipase levels returned to baseline in MET^-/-^ and MET^fl/fl^ mice 48 h post-injury. However, neutrophil infiltration persisted to a significantly greater extent in the MET^-/-^ mice 48 h post-injury, and TUNEL staining detected a 1.3-fold increase in cell death in MET^-/-^ mice relative to MET^fl/fl^ mice at 1 h, increasing to 5.6-fold by 48 h post-injury. Similarly, increased macrophage infiltration and acinar cell proliferation were detected in cerulein treated MET^-/-^ mice relative to cerulein treated MET^fl/fl^ mice at both time points (Fig 4C and S3 Fig). The increased proliferation, cell death and macrophage infiltration observed in MET^-/-^ mice 1 h following cessation of the cerulein treatment, suggested to us that these compensatory responses to pancreatic injury commenced soon after the initial cerulein injection, and implies that MET ordinarily plays an important role in the mechanisms responding to pancreatic tissue injury.

Quantitative RT-PCR (qRT-PCR) detected pronounced upregulation of the proinflammatory mediators TNFα, and RelA in MET^-/-^ mice and MET^fl/fl^ relative to control saline treatments (Fig 4D). Of note, RelA and TNFα levels were consistently higher in MET^-/-^ mice relative to floxed animals 1 h following cerulein treatment. Additionally, IL-1β levels were increased to comparable levels in MET^-/-^ and MET^fl/fl^ mice 1 h following treatment. However, high IL-1β and TNFα levels persisted in MET^-/-^ mice 48 h following treatment. The increase in macrophage infiltration detected in cerulein treated MET^-/-^ mice correlated directly with increased expression of HGF and EMR1, EGF-like module-containing mucin-like hormone receptor-like 1 also known as F4/80, particularly at 48 h following cerulein treatment. Under injurious conditions, endoplasmic reticulum (ER) stress induces the unfolded protein response (UPR), a signaling cascade critical for re-establishing acinar cell homeostasis [28]. Spliced X-box binding protein 1 (sXBP1) acts as an early adaptive response to ER stress to protect acinar cells from ensuing damage and is essential for sustaining the secretory ability of acinar cells [29–31]. However, failure to regain acinar cell homeostasis results in UPR mediated activation of late response cell death components, including the transcriptional factor C/EBP homologous protein 10 (CHOP-10) [32]. The significant increase in sXBP1 expression at 1 h and 48 h detected in the MET^-/-^ mice relative to MET^fl/fl^ mice is envisioned to be an adaptive response to injury in the absence of MET activity (Fig 4D). As expected, no significant change in CHOP-10 expression was noted between MET^-/-^ mice relative to MET^fl/fl^ mice, consistent with the acute nature of the injury. IHC confirmed that MET protein expression was dramatically reduced in MET^-/-^ versus MET^fl/fl^ mice consistent with the acinar-specific MET knockout (data not shown).

### Increased acinar loss, inflammation and collagen deposition in MET^-/-^ mice following recurrent, cerulein injury

**Fig 5:**
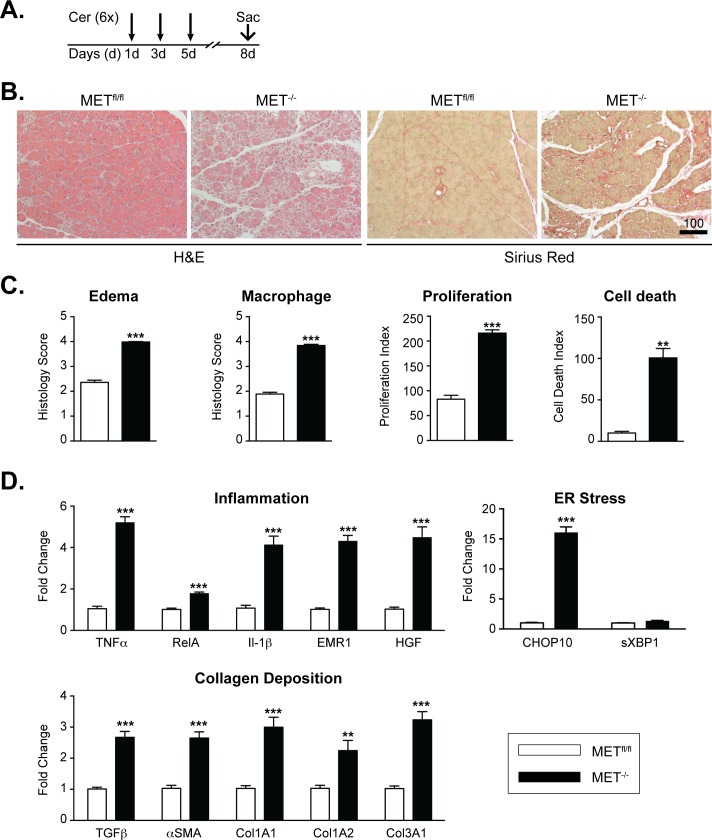
MET is critical for acinar regeneration following recurrent cerulein induced injury. A, MET^fl/fl^ and MET^-/-^ mice were injected with six, hourly-spaced intraperitoneal injections of cerulein, on alternate days for three days and allowed to recover for 72 h. B, Pancreatic sections were processed for H&E and Sirius Red staining to detect tissue morphology and collagen deposition, respectively. C, Morphometric analyses of edema, macrophage infiltration, acinar cell death and proliferation are presented as mean ± SEM values from 4–5 mice per time point Student’s t-test t, **, p < 0.01; ***, p < 0.001). D, qRT-PCR confirmed relative changes in transcript levels in cerulein treated MET^fl/fl^ and MET^-/-^ mice as indicated (Student’s t-test, **, p < 0.01; ***, p < 0.0010).

Our data implies a requirement for MET in the regeneration of the exocrine pancreas following mild acute injury. To determine whether MET is critical for acinar regeneration following recurrent injury, we challenged MET^-/-^ and MET^fl/fl^ mice with repetitive cerulein injections. In this model, mice were injected with six, hourly-spaced intraperitoneal injections of cerulein, on alternate days for three days to perpetuate a microenvironment of severe inflammation and collagen deposition [3] (Fig 5A). H&E and Sirius Red staining revealed pronounced edema associated with significant loss of acinar cells and collagen deposition in MET^-/-^ mice relative to wild type animals (Fig 5B). These changes were associated with increased cell death, cell proliferation and macrophage infiltration in MET^-/-^ mice compared with control mice (Fig 5C and S4 Fig). qRT-PCR detected pronounced upregulation of several proinflammatory mediators in MET^-/-^ mice relative to MET^fl/fl^ including TNFα, RelA and IL-1β. The increased macrophage infiltrate detected in cerulein treated MET^-/-^ mice correlated directly with increased expression of the macrophage marker EMR1, as well as increased pancreatic expression of HGF (Fig 5D). qRT-PCR analysis also detected significant elevation of CHOP-10 expression, a late response marker for ER stress, but not sXBP1 (Fig 5D). Of note, MET deletion resulted in significant upregulation in the expression of α-smooth muscle actin (αSMA) (a marker for pancreatic stellate cell activation) and Transforming Growth Factor β (TGFβ)—a potent pro-fibrotic cytokine—relative to MET^fl/fl^ mice. TGFβ, has been implicated in the pathogenesis of pancreatic fibrosis through the activation of pancreatic stellate cells, which in turn produce several extracellular matrix components, including types 1 and 3 collagen [33]. Consistent with this, qRT-PCR confirmed that the increase in collagen deposition detected in MET^-/-^ mice correlated with increased COL1A1, COL1A2 and COL3A1 expression (Fig 5D). As expected, no neutrophil (Ly6G^+^) infiltrates were detected (data not shown) and serum lipase levels were directly comparable and tested within normal range between MET^fl/fl^ and MET^-/-^ mice (S4 Fig).

### MET regulates acinar renewal and dampens macrophage infiltration following high alcohol intake

**Fig 6:**
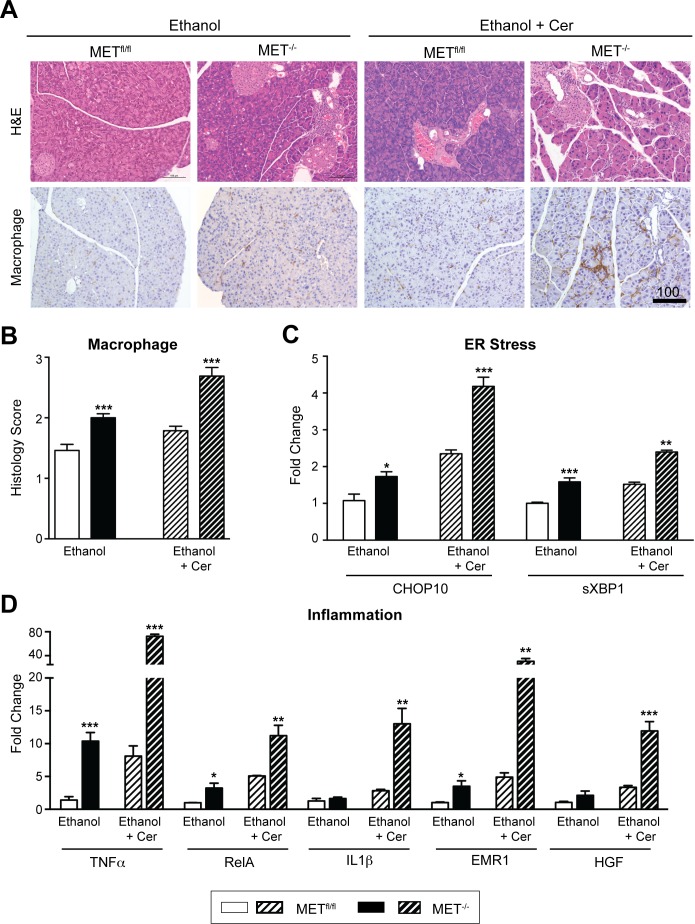
MET regulates acinar renewal and dampens macrophage infiltration following chronic alcohol consumption. A, Representative H&E and macrophage stained sections from MET^fl/fl^ and MET^-/-^ mice fed alcohol ± recurrent cerulein (Cer) induced injury are shown (Scale, microns). B, Morphometric analyses showed increased macrophage infiltration in MET^-/-^ mice (multiple t-test, ***, p < 0.001). C & D, qRT-PCR confirmed relative changes in indicated transcript levels in MET^fl/fl^ and MET^-/-^ mice (multiple t-test, *, p < 0.05; **, p < 0.01; ***, p < 0.001).

Alcohol remains a significant risk factor for pancreatitis in humans. However by itself, heavy alcohol consumption in humans and rodent models does not cause substantial pancreatic damage. Rather alcohol is proposed to function as an etiologic sensitizer, rendering acinar cells susceptible to injury from external stressors or genetic factors [34,35]. Indeed, the combination of alcohol feeding for 14–16 weeks with repeated administration of cerulein in mice was previously reported to result in ethanol-dependent pancreatic collagen deposition, a histologic endpoint of CP [36]. Our data showing that MET is critical for acinar renewal following cerulein-induced mild and recurrent acute injury, prompted us to examine the effect of chronic injury in MET^-/-^ mice following high alcohol intake, in the absence or presence of the exacerbating effects of cerulein. We reasoned that if MET signaling is critical for acinar renewal, we would detect increased acinar death, and chronic inflammation with collagen deposition in MET^-/-^ mice following high alcohol intake and cerulein treatment. To test this, MET^fl/fl^ and MET^-/-^ mice were fed Lieber-DeCarli diets containing 3.5% (v/v) alcohol for 8 weeks. Two cohorts of mice were subjected to repeated episodes of recurrent injury elicited by 3 weeks of alternate, daily i.p. injections of cerulein or saline as a control. Pancreatic morphology and MET expression was examined 3 weeks following the last injection, a time at which fibrosis has been shown to develop in the alcohol-cerulein model [36]. All experimental groups of mice consumed comparable amounts of the liquid diet (data not shown) and exhibited equivalent weight gain (S5 Fig). Serum lipase levels were directly comparable between MET^fl/fl^ and MET^-/-^ mice and tested within normal range (S5 Fig), consistent with the chronic nature of the injury. No MET staining was detected in MET^-/-^ acini consistent with a targeted acinar knockout (data not shown). Morphometric analyses detected a significant preponderance of macrophage (Fig 6A and 6B), T (CD3^+^) and B cell (CD79B^+^) infiltration (data not shown) in the pancreata of MET^-/-^ fed alcohol, particularly in MET^-/-^ mice fed alcohol and co-treated with cerulein, relative to MET^fl/fl^ mice. No neutrophil (Ly6G^+^) infiltrates were detected in MET^-/-^ and MET^fl/fl^ mice consistent with a chronic inflammatory response (data not shown). Moreover, qRT-PCR confirmed significant increases in the expression of the ER stress marker sXBP1 and to a greater extent CHOP-10, in mice deficient in MET, consistent with the chronic nature of the injury (Fig 6C). Consistent with this, qRT-PCR detected pronounced upregulation of the proinflammatory mediators TNFα, RelA and IL1β, as well as increased expression of the macrophage marker EMR1 and HGF, in MET^-/-^ mice relative to MET^fl/fl^ (Fig 6D). Thus MET loss in acinar cells is associated with a chronic inflammatory response and ER stress following ethanol-associated injury.

### MET knockout activates collagen deposition following chronic alcohol intake

**Fig 7:**
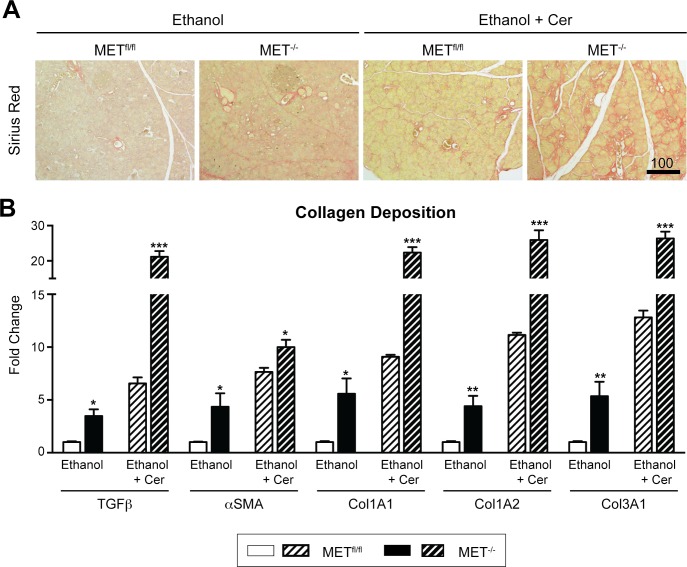
MET deletion caused increased collagen deposition. A, Sirius Red staining confirmed increased collagen deposition in MET^-/-^ relative to MET^fl/fl^ mice fed alcohol ± recurrent cerulein (Cer) induced injury are shown (Scale, microns). B, qRT-PCR confirmed relative changes in transcript levels in alcohol fed MET^-/-^ ± cerulein (Cer) mice for mediators of pancreatic stellate cell activation and collagen deposition (αSMA, TGFβ, COL1A1, COL1A2 and COL3A1) (multiple t-test, *, p < 0.05; **, p < 0.01; ***, p < 0.001).

Studies on pancreatitis patients and using rodent models of pancreatic injury indicate that collagen deposition in the pancreas is a common consequence of severe and chronic inflammation, primarily through macrophage secreted cytokines to promote pancreatic stellate cell activation [37]. We examined whether the heightened inflammatory response observed in the pancreas of MET^-/-^ mice following ethanol-associated injury correlated with changes in collagen deposition. As expected, we detected pronounced collagen deposition in MET^-/-^ pancreas versus MET^fl/fl^ mice as assessed by Sirius Red staining, particularly in MET^-/-^ mice that were fed alcohol and co-treated with cerulein (Fig 7A). The increased expression of αSMA and TGFβ in alcohol-treated MET^-/-^ mice, and to a greater extent in alcohol fed MET^-/-^ mice co-treated with cerulein, correlated strongly with increased expression of the extracellular matrix components Col1A1, Col1A2 and Col3A1 (Fig 7B). Thus, we identified several novel targets downstream of acinar MET signaling important for tissue inflammation and collagen deposition whose expression is altered in response to alcohol-associated injury and/or MET expression.

## Discussion

Here we show that MET levels are low in the normal adult pancreas, but are elevated in ductal and acinar cells in human CP specimens. Hence, it is conceivable that the HGF/MET axis constitutes an organotrophic signaling mechanism against organ injury. To test this directly, we selectively deleted MET in adult murine acinar cells and assessed their response to four models of pancreatic injury and regeneration that differed in the severity of injury as well as the insult agent. We report that the loss of acinar MET enhanced inflammation and was accompanied with a delay in the requisite acinar tissue remodeling and recovery, suggesting that MET has a protective role against the injury in acinar cells in general. Our data are significant for highlighting the importance of MET during intrinsic acinar tissue repair in response to acute and chronic pancreatic injury.

Acinar cells that survive cerulein-induced injury are capable of regenerating the pancreatic exocrine tissue. In MET^fl/fl^ mice we detected two waves of cell death following acute cerulein-induced injury—the first immediately following injury with the second phase peaking 72 h following injury. We hypothesize that initial phase of cell death reflects loss of acinar cells due to the injury, whereas the second phase of cell death likely represents a compensatory mechanism to restore proper pancreatic cell mass by pruning surplus acinar cells generated due to robust proliferation during the regenerative phase (see Fig 2B). Following repetitive cerulein-induced injury, we show that the pronounced inflammatory response typically detected in injured MET^-/-^ mice associates with a strong stromal reaction, evidenced by up-regulation of profibrotic genes (TGFβ and αSMA), increased expression of fibrotic collagens (Col1A1, Col1A2, Col3A1) and increased collagen deposition. Interestingly, serum lipase levels, a measure of immediate pancreatic damage, were directly comparable between the two MET genotypes following acute and repetitive cerulein-induced injury, suggesting that MET signaling does not directly affect the magnitude of acinar cell injury. Rather, acinar MET signaling contributes to the resolution of inflammation and the promotion of tissue repair following acute and repetitive cerulein induced injury.

Emerging evidence suggests that an early and sustained activation of inflammatory signaling in acinar cells is responsible for the intense local and systemic inflammatory responses detected during pancreatic injury [26,38]. TNFα, a major inflammatory cytokine produced and secreted during acute and CP from acinar cells and invading leukocytes, activates pancreatic NF-ĸB, a key transcriptional regulator of the expression of inflammatory molecules. Increased TNFα levels correlated directly with increased cytokine expression and severity of acute pancreatitis in a mouse model of pancreatitis [39,40]. MET was recently shown to be a direct NF-ĸB transcriptional target [41,42]. Thus it is tempting to speculate that the increase in TNFα signaling in injured pancreatic tissue following acute, repetitive and chronic injury functions to promote MET expression as a wound healing response. Consistent with this tenet, we also noted upregulation of HGF expression in MET^-/-^ mice, which may represent a compensatory mechanism for acinar injury in the absence of MET signaling. Alternatively, the increase in HGF expression may be a consequence of the pronounced macrophage infiltrate noted in mice deficient in MET following acute and repetitive injury, since macrophage derived HGF is critical for wound repair.

The association between alcohol and CP has been recognized for well over a century. Recent population studies reported that alcohol increased the risk of CP 2–4 fold, depending on the dose and duration of alcohol exposure. By itself, heavy alcohol consumption in humans and rodent models does not cause substantial pancreatic damage. Rather alcohol is proposed to function as an etiologic sensitizer, rendering acinar cells more susceptible to injury from external stressors or genetic factors [34,35]. Our data showing that alcohol promotes increased collagen deposition (a measure of tissue fibrosis) in the pancreas of MET^-/-^ mice relative to MET^fl/fl^ animals. One possible explanation for this observation is that the increased expression of TNFα in the MET^-/-^ mice may result in stellate cell activation and proliferation [43]. Alternatively, the increased infiltration of macrophages in the MET^-/-^ mice, may promote stromal changes that result in collagen deposition [36,37]. When alcohol fed mice were exposed to cerulein induced acinar injury, we detected a profound increase in macrophage infiltration, collagen deposition in the periacinar region of the pancreatic parenchyma with significant upregulation of proinflammatory, profibrotic and extracellular matrix markers in MET^-/-^ mice relative to MET^fl/fl^ animals, and animals fed alcohol alone. Consistent with our earlier data indicating that acinar repair is initiated following injury in MET^-/-^ mice, serum lipase levels were directly comparable between experimental cohorts. Our data are consistent with the hypothesis that when the self-renewing capacity of the exocrine pancreas is overwhelmed, inflammation ensues resulting in irreversible tissue damage. It is tempting to speculate that the loss of MET in acinar cells appears to lower the threshold at which irreparable damage occurs.

The ER is a multifunctional organ responsible for the synthesis and correct folding of proteins destined for secretion. Pancreatic acinar cells maintain a high level of protein secretion, and as such the maintenance acinar ER homeostasis is critical. Pancreatic ER stress or the unfolded protein response (UPR) results in the activation of the early protective factor sXBP1 as well as pro-apoptotic signals such as CHOP10 [44,45]. Studies using genetically engineered cells and knockout models suggest that sXBP1 functions as an early adaptive component to protect acinar cells from injury, thereby restoring the secretory capacity of the injured exocrine pancreas [30] and limiting the deleterious effects of the pro-apoptotic factor CHOP10. When the level of misfolded protein levels in the ER remains unresolved, increased expression of CHOP10 ensues leading to programmed cell death [46]. Thus the balance between cell proliferation and apoptosis is regulated in part by increased sXBP1 versus CHOP10 expression respectively. The higher level of sXBP1 detected in MET^-/-^ mice relative to MET^fl/fl^ controls may indicate a compensatory mechanism for driving acinar regeneration in the absence of MET. Consistent with this, CHOP10 remained directly comparable between MET^-/-^ and MET^fl/fl^ mice under these conditions. Thus, in the absence of MET, reparative ER stress responses predominate over pro-apoptotic pathways in an effort to elicit the resolution of inflammation and promote the tissue repair. Conversely, CHOP10 levels were ~15 fold higher in MET^-/-^ mice relative to MET^fl/fl^ animals following recurrent cerulein-induced injury, correlating closely with acinar cell loss, sustained inflammation and collagen deposition. sXBP1 levels remained directly comparable between both experimental groups under these conditions. Of note, we show increased sXBP1 and CHOP10 expression following alcohol-associated injury in the absence or presence of cerulein in MET^-/-^ mice relative to the wild-type controls. Additional studies will be required to fully understand how MET signaling may affect ER stress responses to favor the regeneration of injured acinar cells.

In summary, pancreatic acinar cells possess a remarkable capacity for regeneration. Here we present a novel role for MET signaling in pancreatic plasticity following acute and chronic models of acinar injury. Our findings are timely and may form the basis for targeted therapeutics designed to promote repair of the exocrine pancreas.

## Supporting Information

S1 Fig Representative immunohistochemistry sections analyzed at the indicated times for tissue injury (edema and cell death), repair (cell proliferation), macrophage and neutrophil invasion in MET^-/-^ mice relative to MET^fl/fl^ mice following acute cerulein injury (Scale, microns). (TIF)

S2 Fig Islet size and number, and serum insulin levels were directly comparable between naïve MET^-/-^ and MET^fl/fl^ mice. (TIF)

S3 Fig Representative pancreatic sections analyzed for histological changes display increased tissue injury (cell death), repair (cell proliferation), macrophage and neutrophil invasion in MET^-/-^ mice relative to MET^fl/fl^ mice in response to acute cerulein injury (Scale, microns). (TIF)

S4 Fig A, Comparable levels of serum lipase were noted in MET^-/-^ and MET^fl/fl^ mice following recurrent cerulein injury. B, Representative pancreatic sections analyzed for histological changes display increased tissue injury (cell death), repair (cell proliferation) and macrophage invasion in MET^-/-^ mice relative to MET^fl/fl^ controls following recurrent cerulein induced injury (Scale, microns).
(TIF)

S5 Fig **All experimental groups of mice exhibited equivalent serum lipase levels (A) and weight gain (B).** Note the comparable drop in weight in MET^-/-^ and MET^fl/fl^ mice treated with cerulein (Cer).
(TIF)

## Abbreviations

AP: acute pancreatitis
αSMA: α-smooth muscle actin
Cer: cerulein
CP: chronic pancreatitis
CHOP10: C/EBP homologous Protein 10
EMR1: EGF-like module-containing mucin-like hormone receptor-like 1
ER: Endoplasmic Reticulum
HGF: Hepatocyte Growth Factor
IHC: immunohistochemistry
i.p.: intraperitoneal
MET: Hepatocyte Growth Factor Receptor
MET^-/-^: conditional MET knockout mouse
NF-ĸB: nuclear factor-ĸB
RelA: v-rel avian reticuloendotheliosis viral oncogene homolog A
sXBP1: spliced X-box binding protein 1
TGFβ: Transforming Growth Factor β
TNFα: Tumor Necrosis Factor α
UPR: Unfolded Protein Response
