# Supplementary figures and images for: The MET Receptor Tyrosine Kinase Confers Repair of Murine Pancreatic Acinar Cells following Acute and Chronic Injury
Source: PLoS One. 2016 Oct 31;11(10):e0165485. doi: 10.1371/journal.pone.0165485 (PMC5087859; doi:10.1371/journal.pone.0165485)

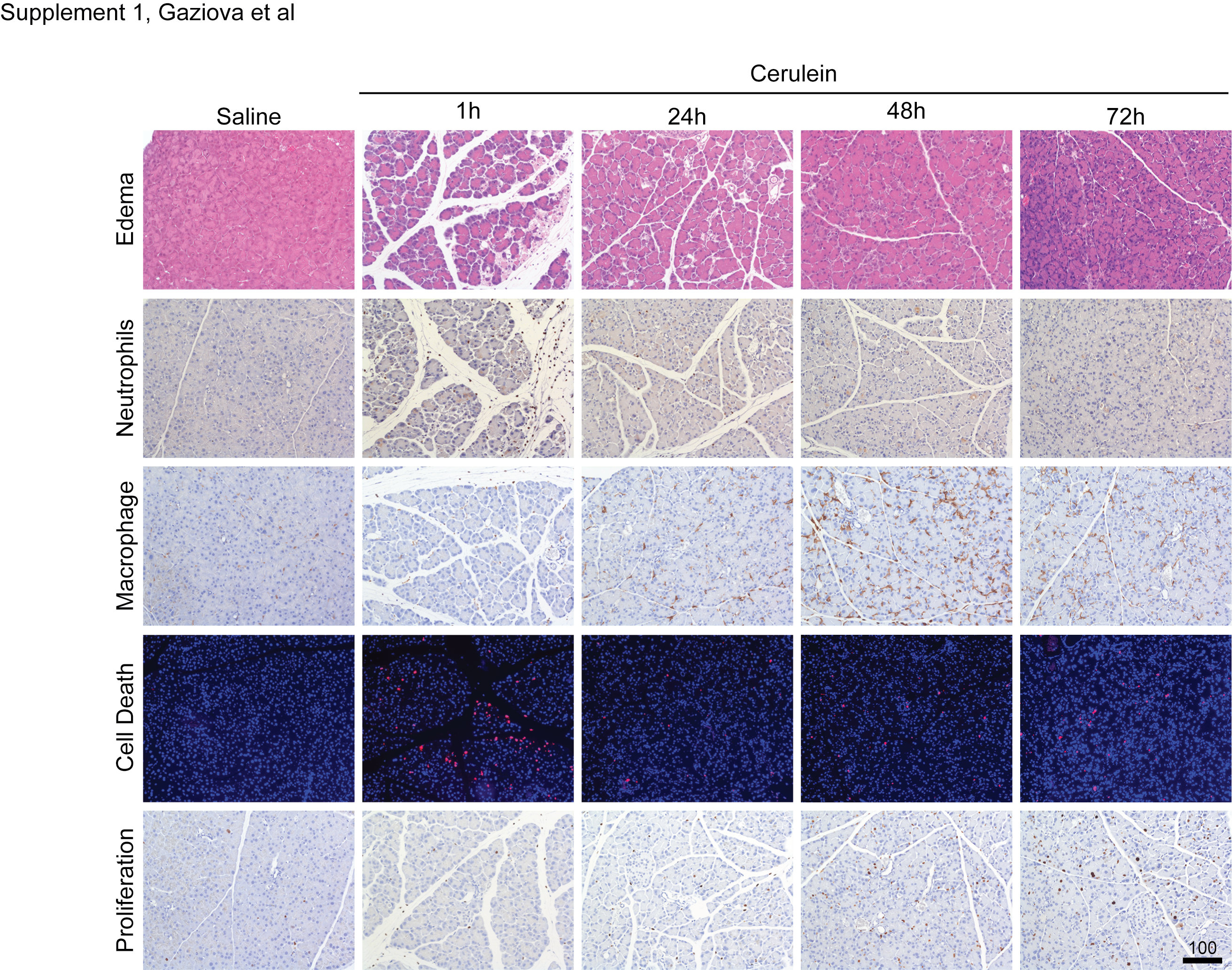

Supplement: S1 Fig — (TIF) [file pone.0165485.s001.tif]

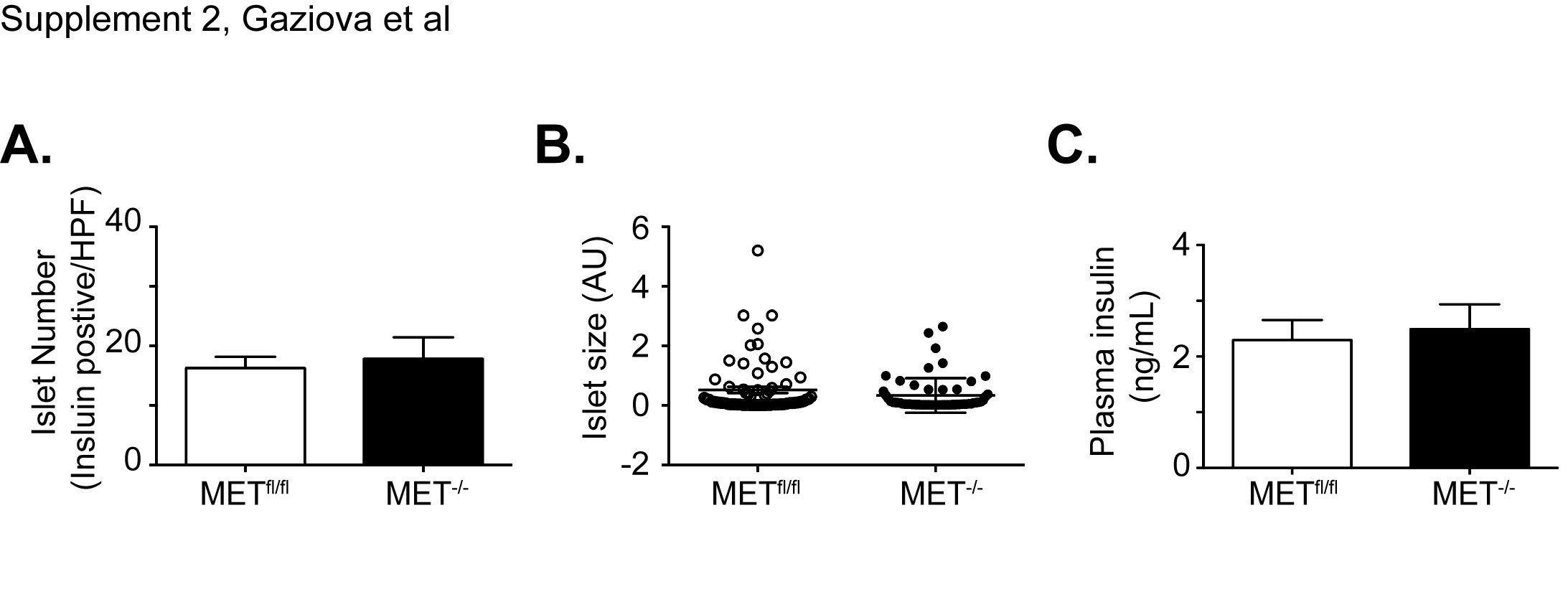

Supplement: S2 Fig — (TIF) [file pone.0165485.s002.tif]

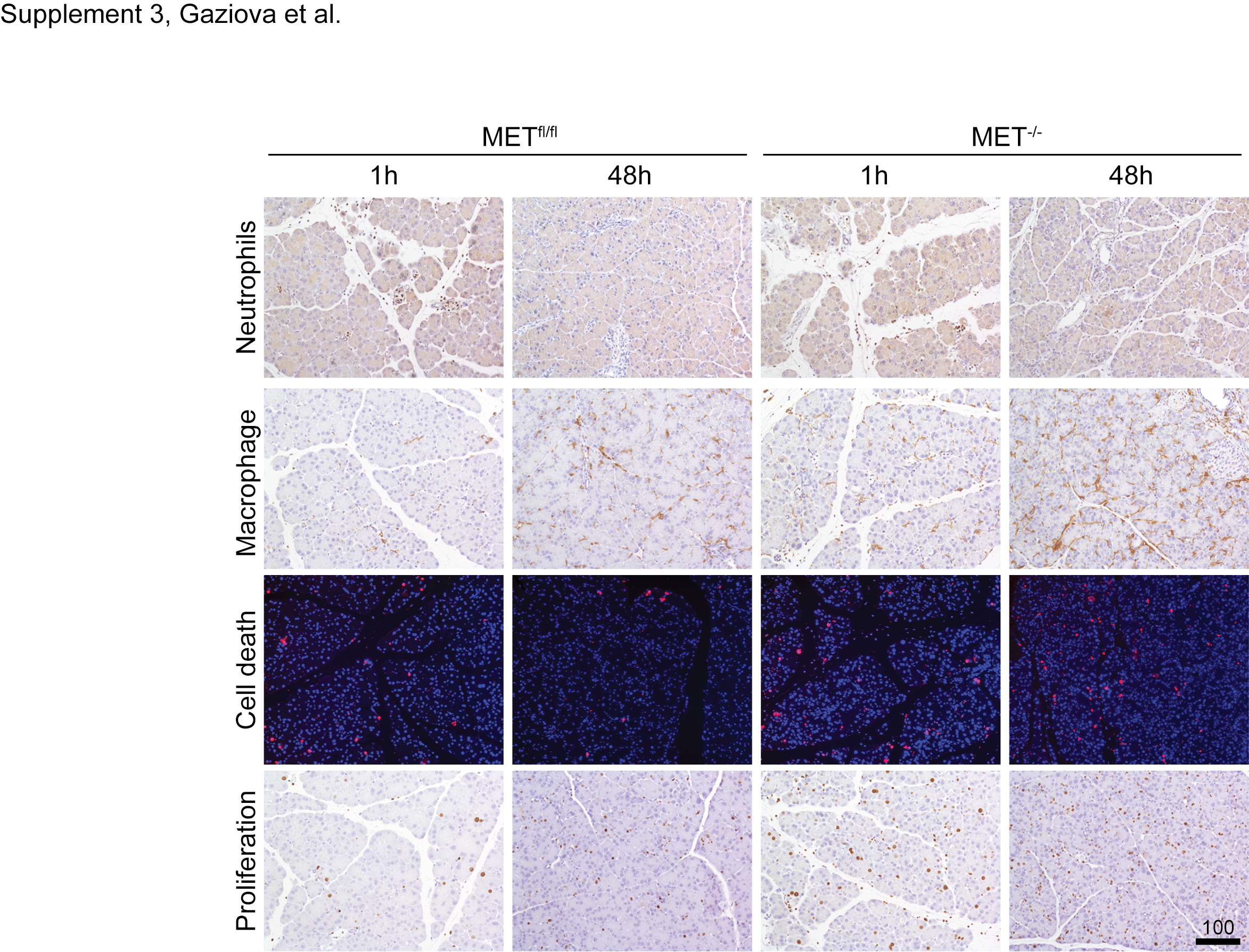

Supplement: S3 Fig — (TIF) [file pone.0165485.s003.tif]

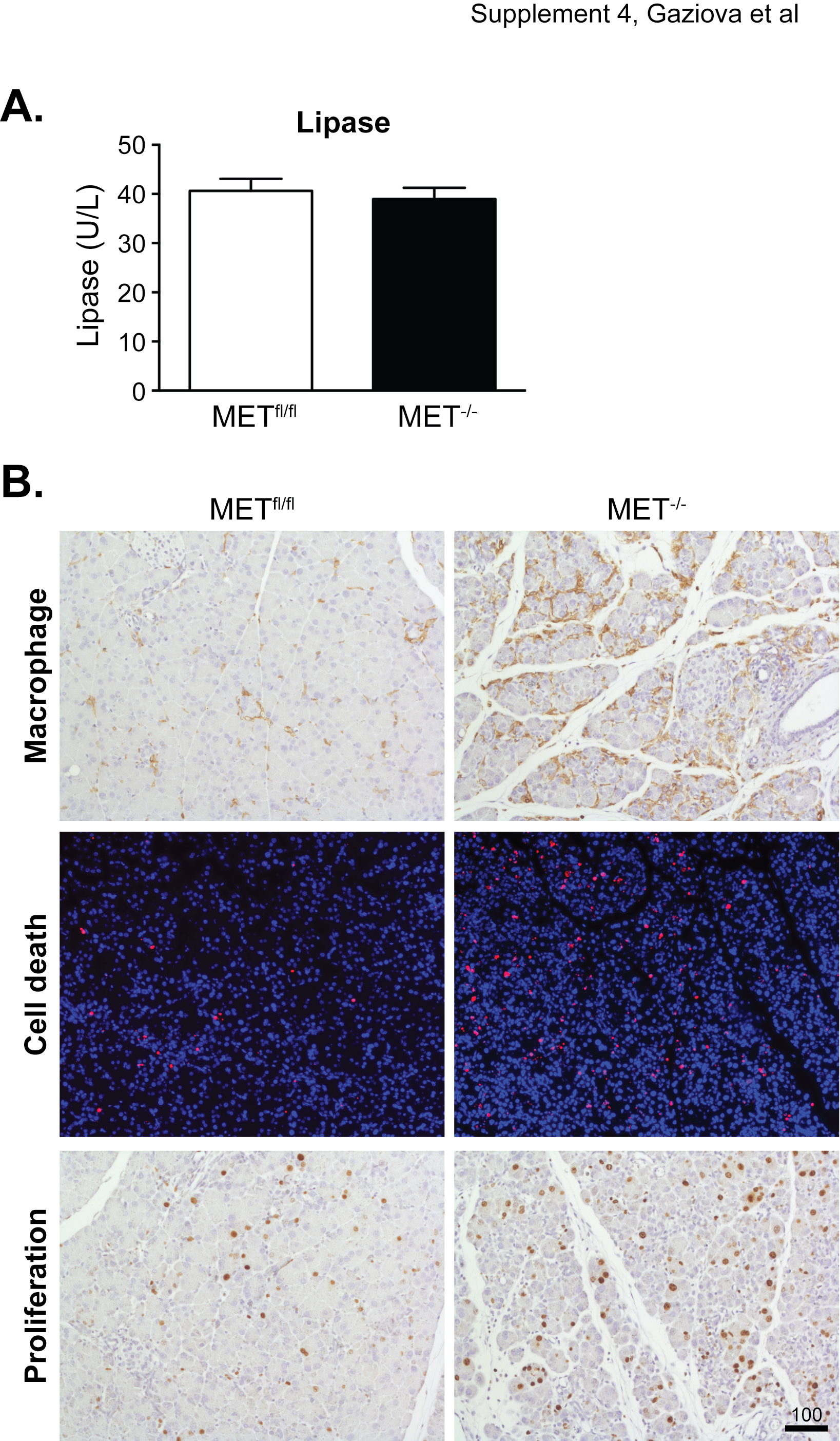

Supplement: S4 Fig — A, Comparable levels of serum lipase were noted in MET-/- and METfl/fl mice following recurrent cerulein injury. B, Representative pancreatic sections analyzed for histological changes display increased tissue injury (cell death), repair (cell proliferation) and macrophage invasion in MET-/- mice relative to METfl/fl controls following recurrent cerulein induced injury (Scale, microns). (TIF) [file pone.0165485.s004.tif]

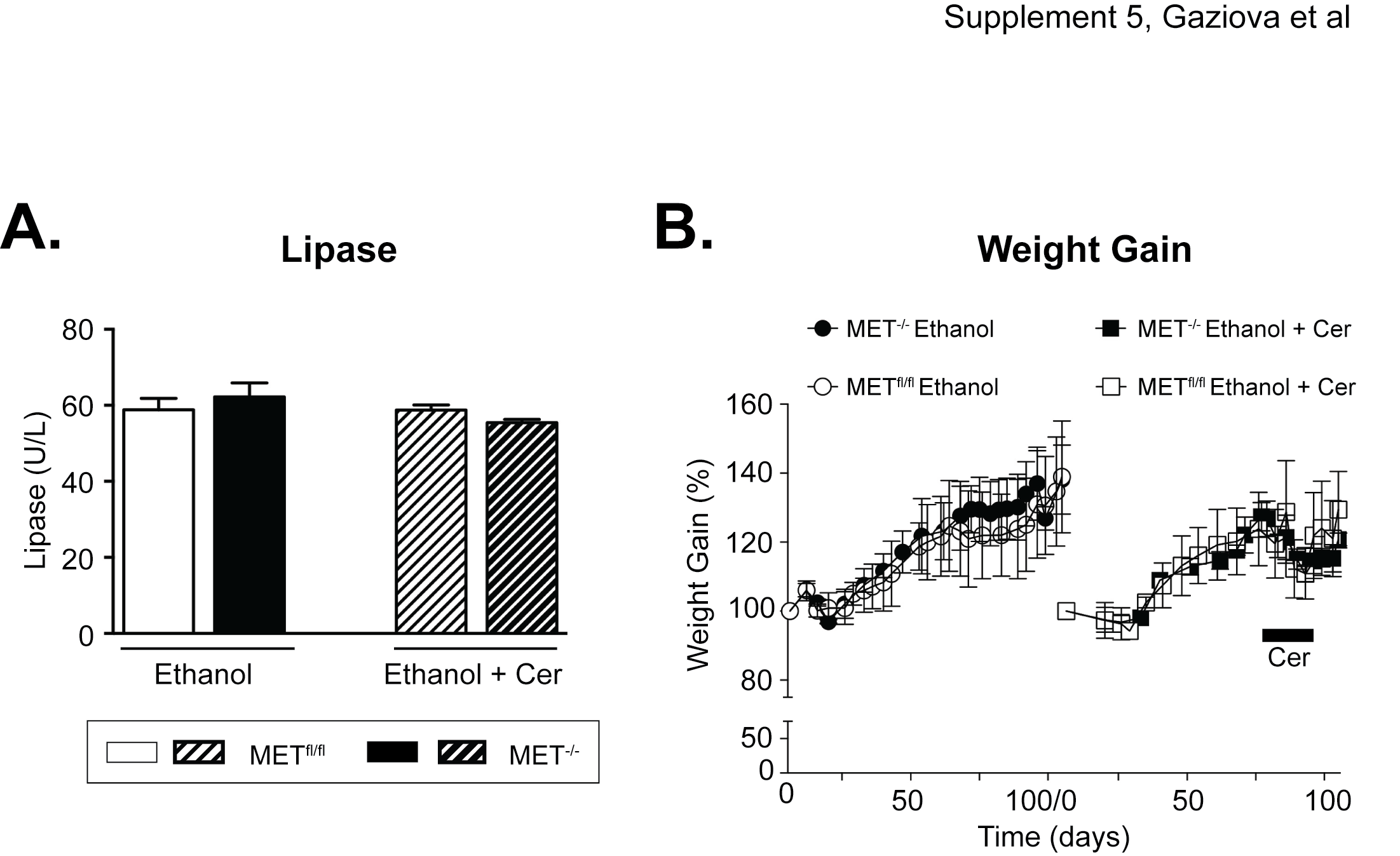

Supplement: S5 Fig — All experimental groups of mice exhibited equivalent serum lipase levels (A) and weight gain (B). Note the comparable drop in weight in MET-/- and METfl/fl mice treated with cerulein (Cer). (TIF) [file pone.0165485.s005.tif]
